# MicroRNA-182 Suppresses HGF/SF-Induced Increases in Retinal Pigment Epithelial Cell Proliferation and Migration through Targeting c-Met

**DOI:** 10.1371/journal.pone.0167684

**Published:** 2016-12-09

**Authors:** Lihua Wang, Feng Dong, Peter S. Reinach, Dandan He, Xiaoting Zhao, Xiaoyan Chen, Dan-Ning Hu, Dongsheng Yan

**Affiliations:** 1 School of Ophthalmology and Optometry, Eye Hospital, Wenzhou Medical University, Wenzhou, Zhejiang, China; 2 State Key Laboratory Cultivation Base and Key Laboratory of Vision Science, Ministry of Health of the People’s Republic of China, Zhejiang Provincial Key Laboratory of Ophthalmology and Optometry, Wenzhou, Zhejiang, China; 3 Tissue Culture Center, the New York Eye and Ear Infirmary, New York Medical College, New York, New York, United States of America; 4 The First Affiliated Hospital of College of Medicine, Zhejiang University, Hangzhou, Zhejiang, China; Duke University, UNITED STATES

## Abstract

As increases in hepatocyte growth factor/scatter factor (HGF/SF) induce retinal pigment epithelial (RPE) migration and proliferation into the vitreous cavity and contribute to proliferative vitreoretinopathy (PVR) development, we determined if changes in miR-182 expression affect such behavioral changes. We found that miR-182 expression was less in PVR clinical samples than in primary RPE cells whereas c-Met was upregulated. Ectopic miR-182 inhibited RPE cell proliferation, cell cycle, and migration. Bioinformatic analysis identified c-Met as a miR-182 target, which was confirmed with the luciferase reporter assay. Transfection of miR-182 into RPE cells induced c-Met downregulation, which led to reduced cell proliferation and migration through declines in p-Akt formation. MiR-182 downregulation along with c-Met upregulation in PVR tissues suggest that these two opposing effects play important roles in PVR development. As ectopic miR-182 expression suppressed RPE cell proliferation and migration, strategies to selectively upregulate miR-182 expression in a clinical setting may provide a novel option to treat this disease.

## Introduction

Proliferative vitreoretinopathy (PVR) is a sight compromising pathological response to either retinal reattachment surgery or ocular trauma. This condition arises from retinal detachment surgery in 5–10% of the cases leading to scarring and inflammation during wound healing [1–5]. These side effects are accompanied by formation of sight compromising epiretinal membranes containing a mixture of different retinal derived cell types. They include retinal pigment epithelial (RPE) cells, glial and Muller cells as well as fibroblasts and activated immune cells that are induced to translocate into the vitreous chamber and elaborate these sight obstructing membranes and inflammation as well as scarring. RPE cells are an important contributor to PVR development [1–6]. The only somewhat effective treatment for this condition is surgical removal of these membranes [4, 7]. However, this procedure is problematic because membranes may reform due to the aforementioned surgical-induced side effects. There are no effective drugs for PVR treatment because the molecular mechanisms underlying PVR remain largely unclear. So it is vital to identify specific drug targets whose modulation can block this pathological process.

MicroRNAs (miRNAs) are highly conserved non-coding small RNA molecules first discovered in *C*. *elegans* in 1993 [8]. Since their discovery, over 2,000 members have been identified in humans. It is estimated that they can regulate 20–30% of the protein-coding genes in the human genome [9, 10]. Their control is elicited by binding to complementary messenger RNA sequences, resulting in post-transcriptional gene silencing and inhibition of protein translation [10]. A single miRNA can downregulate multiple targets, which often belong to the same metabolic or signaling pathway. Such effects account for their importance in controlling a multitude of responses essential for tissue function. Their involvement includes controlling gene expression contributing to cell proliferation, differentiation, apoptosis and development [10, 11]. On the other hand, dysregulated miRNA expression has been identified in various human diseases such as cancer [12].

In a number of tissues, miRNAs may have a pivotal role in regulating tumor progression by modulating c-Met gene expression levels [13, 14]. C-Met is highly expressed in the RPE cells and is a viable gene target to control RPE involvement in PVR development [15, 16]. This is evident since in a retinal detachment mouse model increases in HGF/SF levels occur eliciting c-Met upregulation followed by increases in RPE migration [17]. One miRNA candidate modulating c-Met expression in RPE cells is miR-34a [18]. Downregulation of c-Met subsequent to miR-34a upregulation suppressed RPE cell proliferation and migration. However, the possible roles of miRNAs in clinical PVR tissue samples have not been evaluated.

In order to increase our chances of identifying viable miRNAs candidates that underlie molecular events contributing to the PVR phenotype, we started by pinpointing miRNAs in other ocular tissues whose modulation affect tumorigenic activity. This approach was taken since during PVR development RPE cells undergo dedifferentiation as they proliferate and migrate. In culling the miRNA candidates, we also took into account that disrupted p53 activity is thought to contribute to the RPE cell dedifferentiation process causing them to become invasive and transition into a myofibroblast phenotype [19–21]. These considerations prompted us to evaluate the role of miR-182 in this process since we previously showed in uveal melanoma cells that declines in p53 expression are associated with dramatic miR-182 downregulation and dedifferentiation leading to tumorous metastatic behaviour [22].

We show here that miR-182 was downregulated in PVR specimens whereas c-Met expression was upregulated as compared to corresponding levels in normal RPE cells. Either upregulating miR-182 in RPE cells or downregulating c-Met expression reduced HGF/SF-induced rises in both RPE cell proliferation and chemotaxis through declines in Akt activation. Furthermore, c-Met was identified as a miR-182 gene target through bioinformatics and functional assays. Altogether, the control of c-Met expression and downstream signalling in RPE by miR-182 suggests that PVR development may be hindered through upregulating miR-182 expression.

## Results

### MiR-182 expression lowering in PVR clinical samples

**Fig 1:**
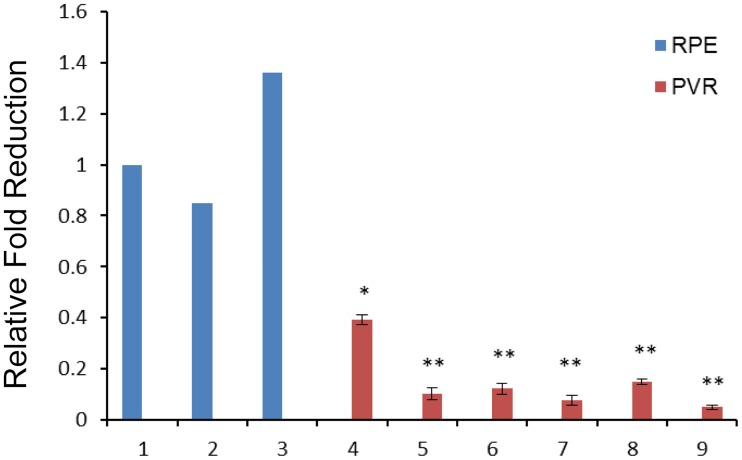
MiR-182 expression is frequently downregulated in PVR specimens. Real-time RT-PCR analysis was performed to detect the expression of miR-182 in six PVR specimens, as well as RPE cells from healthy donors. The average value for miR-182 in isolated RPE cells from three donors was set at 1, and the relative amount of miR-182 in the specimens was plotted as fold reduction. 1–3: normal RPE cells; 4–9: PVR specimens. U6 snRNA was used as an internal control. *: Differences in miR-182 expression between RPE cells and PVR specimens were significant (*: *P* < 0.05, **: *P* <0.01).

To evaluate if miR-182 expression levels are abnormal in PVR pathogenesis, we used Real-time RT-PCR to compare miR-182 levels in PVR clinical samples with those in isolated RPE cells from three healthy donors. In six PVR clinical specimens, miR-182 levels were significantly downregulated compared to those in RPE cells used as normal controls (Fig 1).

### Reciprocal relationship between RPE miR-182 expression and cell proliferation

**Fig 2:**
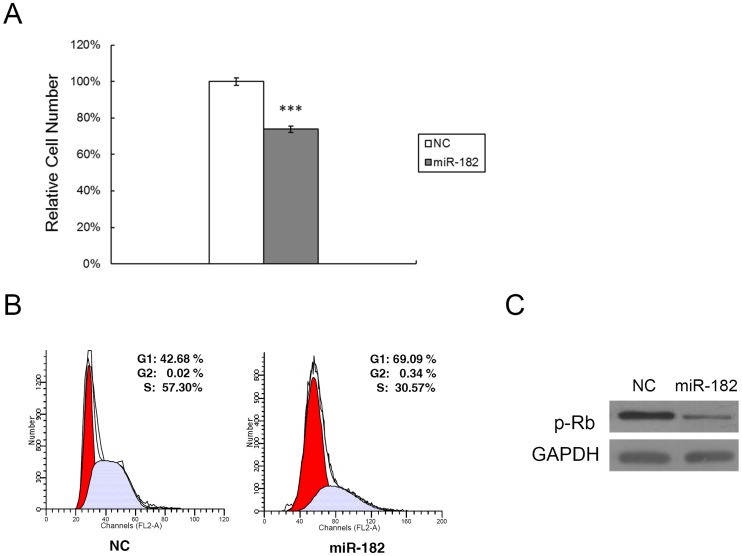
Ectopic miR-182 expression induces RPE cell G1 arrest and inhibits cell proliferation. (A) MTS cell proliferation assay was carried out as indicated after lipofectamine transfection of RPE cells with either miR-182 (50 nM) or a negative control (NC). The data are expressed as the mean value ± SEM of the results obtained from triplicates in one experiment. Results represent those obtained in three separate experiments. (B) RPE cells were collected 48 hours after transfection with miR-182 or NC, stained with propidium iodide and analyzed by flow cytometry. Ten thousand cells were evaluated in each sample. The most representative results in three independent experiments are depicted. (C) miR-182 regulated cell cycle-related proteins that are important for cell cycle G1 phase progression and G1/S transition. RPE cells were transfected with miR-182, or a negative control. Cell lysates were prepared and used for Western blot analysis with phosphorylated Rb (p-Rb) antibody. GAPDH was used as a loading control. ***: Differences in cell proliferation between miR-182 and NC transfected cells were significant, *P* < 0.001.

The marked miR-182 decrease in PVR clinical samples prompted us to determine in PVR whether or not such a decline may contribute to increases in RPE proliferation occurring in this disease process. As expected, miR-182 transfection caused a remarkable inhibition of RPE cell proliferation compared with that in other cells transfected instead with its irrelevant negative control (Fig 2A). We also determined if this decline is consistent with an effect on cell cycle progression. Cell cycle mapping indicated that the miR-182-induced decline in cell proliferation is consistent with more pronounced G1 cell cycle arrest at the G1/S checkpoint in these cells (Fig 2B). We next determined if this decline in cell cycle progression caused by ectopic miR-182 expression is consistent with an expected decline in retinoblastoma protein (p-Rb) phosphorylation status, which reduces cell transit through the G1/S checkpoint. The results shown in Fig 2C are consistent with this expectation since the p-Rb phosphorylation status declined.

### Restoration of miR-182 inhibits RPE cell migration

**Fig 3:**
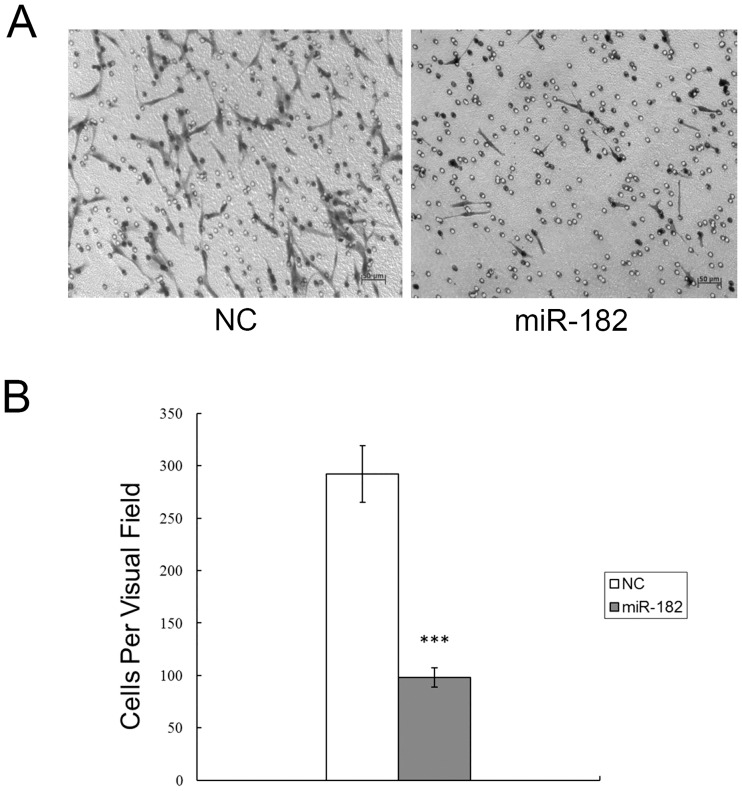
Ectopic miR-182 expression inhibits RPE cell migration. (A) The number of RPE cells that had migrated through the culture insert pores was quantified by counting five independent visual fields using a 20X microscope objective. (B) Results represent those obtained in three experiments. ***: Differences in cell migration between miR-182 and negative control transfected cells were significant, *P* < 0.001.

To further explore the physiological relevance of a decline in miR-182 expression in PVR clinical samples, we determined if the migration rates of miR-182 transfected RPE cells were different from those in their irrelevant negative controls. As shown in Fig 3A, HGF/SF-induced migration decreased more in miR-182 transfected cells than in negative control transfected cells. Fig 3B provides a summary plot and analysis of these results.

### C-Met is a miR-182 gene target

**Fig 4:**
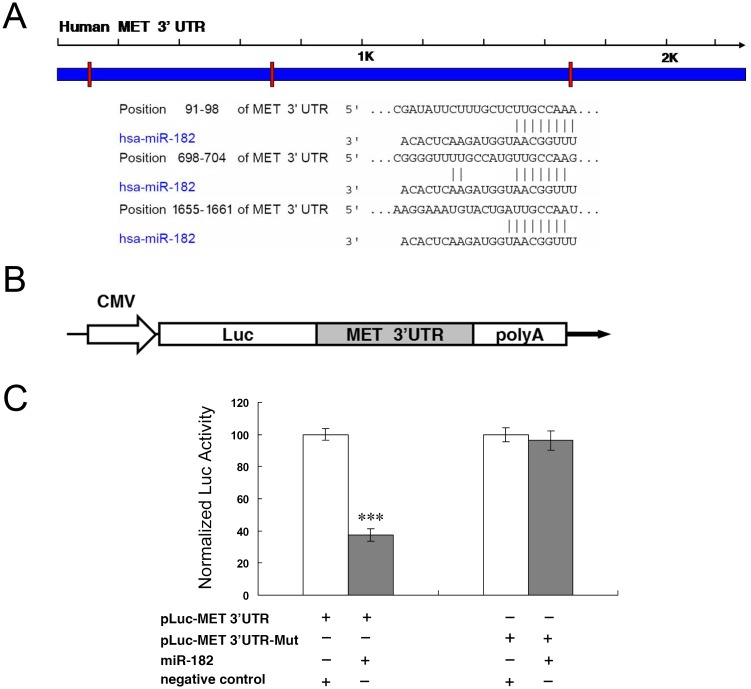
C-Met is a miR-182 gene target. (A) Specific locations of the binding sites are marked with red color and c-Met 3' UTR are marked with blue color. Alignment between the predicted miR-182 target sites and miR-182, the conserved 7–8 bp “seed” sequence for miR-182:mRNA pairing is indicated. (B) Diagram depicting the pMIR luciferase reporter constructs, containing a CMV promoter, which was utilized to verify the putative miR-182 binding sites. (C) HEK293 cells were co-transfected with miR-182, pLuc-MET 3' UTR, along with a pRL-SV40 reporter plasmid. After 24 h, the luciferase activity was measured. Values are presented as relative luciferase activity after normalization to *Renilla* luciferase activity. Results represent those obtained in three separate experiments. ***: Differences in luciferase activity between miR-182 and negative control transfected cells were significant, *P* < 0.001.

To identify miR-182 gene targets, we searched public databases using TargetScan (http://www.targetscan.org). C-Met was selected as a pertinent gene target whose downregulation may account for why miR-182 transfection reduced cell proliferation and migration. Our decision to make this assessment stemmed from an earlier report in which it was shown in a mouse retinal detachment model that increases in vitreous chamber HGF/SF content are associated with c-Met upregulation and activation [17]. We identified with bioinformatic analysis the various target sequences in the 3' UTR of c-Met gene (Fig 4A). In order to test if miR-182 directly targets the candidate gene, we cloned the entire wildtype 3' UTR of c-Met gene into a luciferase gene reporter vector shown in Fig 4B. We then transfected each time the same reporter construct (pLuc-MET 3' UTR) into HEK293 cells, along with either miR-182 or an irrelevant negative control. The luciferase gene reporter activity assays at 24 h post-transfection demonstrated that miR-182 dramatically suppressed this activity (Fig 4C). On the other hand, mutations of all the binding sites in the c-Met gene attenuated the suppression of such activity caused by miR-182 (Fig 4C). These results demonstrate that miR-182 directly targets the c-Met gene through binding to its 3' UTR, which is requisite for miR-182 to suppress c-Met activity.

### C-Met downregulation inhibits RPE cell proliferation and migration

**Fig 5:**
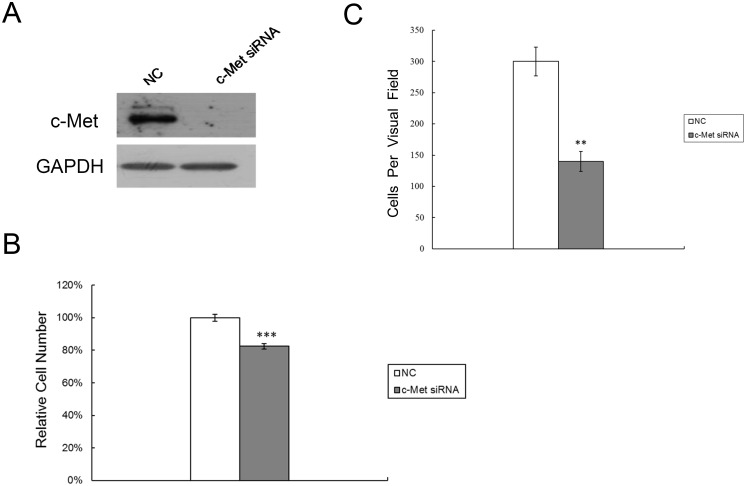
Downregulation of c-Met inhibits RPE cell proliferation and migration. (A) Western blot analysis was performed to detect c-Met expression after lipofectamine transfection of RPE cells with either c-Met siRNA (50 nM) or a negative control (NC). (B) MTS cell proliferation assay was carried out on days 3 after transfection with c-Met siRNA (50 nM) or NC. (C) RPE cells transfected with c-Met siRNA or NC, were quantified for migratory studies using transwell assay. Results represent those obtained in three experiments. **: Differences in cell migration between c-Met siRNA and NC transfected cells were significant, *P* <0.01. ***: Differences in cell proliferation between c-Met siRNA and NC transfected cells were significant, *P* <0.001.

Since c-Met is a direct target of miR-182, we next determined if a decline in c-Met expression had corresponding effects on cell proliferation and migration. For this purpose, c-Met specific siRNA was used to knockdown c-Met expression in RPE cells. Fig 5A results show that c-Met siRNA transfection was effective since c-Met protein expression decreased dramatically. In these transfected cells, MTS assay results show that increases in cell number at 72 h were significantly less than those in cells transfected instead with a negative control (Fig 5B). Similarly, c-Met siRNA transfection also significantly hampered HGF/SF-induced increases in migration (Fig 5C).

### C-Met downregulation reduces rises in RPE proliferation and migration through blunting of linked cell signalling activation

**Fig 6:**
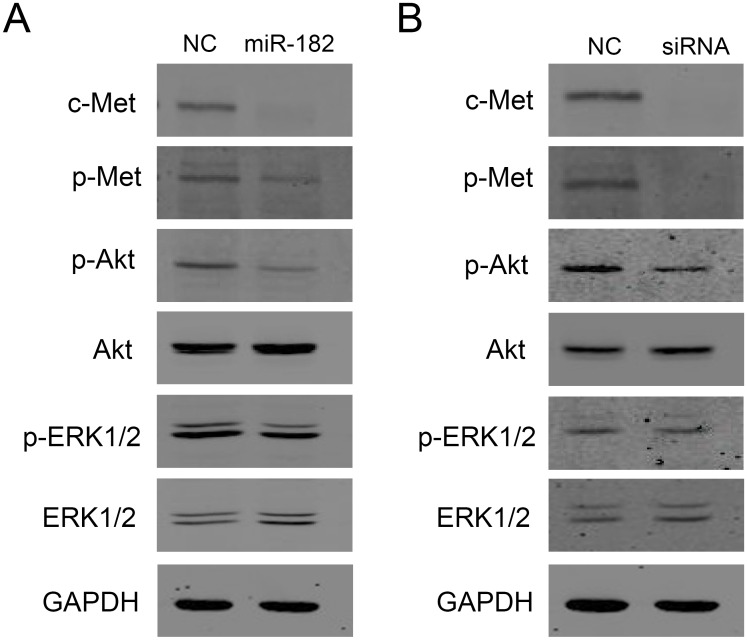
Ectopic miR-182 expression or c-Met siRNA transfection downregulates multiple cell signaling pathways. RPE cells were transfected with miR-182 or c-Met specific siRNA and then analyzed by Western blotting. (A) miR-182 downregulated expression of c-Met, p-Met and p-Akt, but not total Akt or ERK1/2. (B) c-Met specific siRNA downregulated expression of c-Met, p-Met and p-Akt, but not total Akt or ERK1/2.

HGF/SF-induced c-Met dimerization and phosphorylation induce downstream responses through a network of interacting signaling pathways transiently phosphorylating kinases in the mitogen activated protein kinase (MAPK) cascade and the PI3K/Akt signaling pathway. To document that downregulation of c-Met by either miR-182 or c-Met specific siRNA transfection in RPE cells has downstream signalling effects accounting for declines in cell proliferation and migration, we determined if such suppression was accompanied by declines in ERK1/2 and Akt phosphorylation status. As shown in Fig 6A, downregulation of c-Met by miR-182 led to significant reductions of both phosphorylated-c-Met and phosphorylated-Akt levels. On the other hand, both total Akt and total ERK1/2 levels were invariant (Fig 6A). Meanwhile, c-Met downregulation by c-Met siRNA transfection also caused remarkable decreases in both phosphorylated-c-Met and phosphorylated-Akt levels (Fig 6B). These results suggest that miR-182 inhibits RPE cell proliferation and migration, at least in part, by targeting c-Met in its downstream PI3K/Akt signaling pathway.

### Reciprocal relationship between c-Met and miR-182 levels in PVR clinical samples

**Fig 7:**
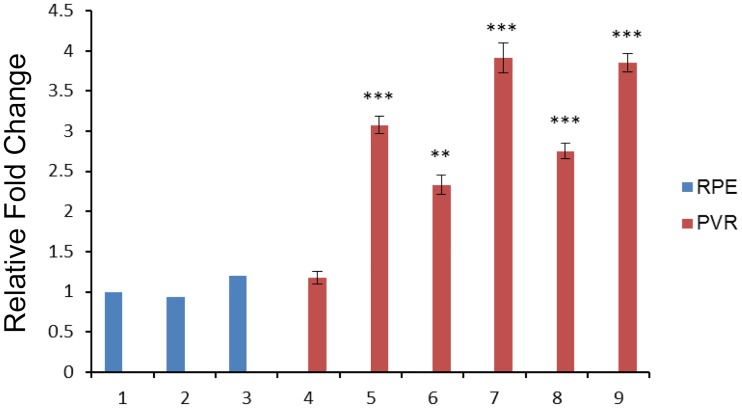
The expression levels of c-Met in PVR specimens were upregulated. The majority of samples examined (#4-#9) showed c-Met upregulation, as compared with RPE cells from healthy donors (#1-#3). *: Differences in c-Met expression between RPE cells and PVR specimens were significant (**: *P* <0.01, ***: *P* <0.001).

As miR-182 levels were lower in PVR clinical samples than those in normal RPE cells whereas miR-182 transfection in RPE cells downregulated c-Met expression, this led us to hypothesize that in PVR clinical samples c-Met gene expression is elevated relative to that in RPE cells. Indeed this prediction was found to be correct since Real time RT-PCR results shown in Fig 7 indicate that c-Met expression was remarkably elevated in all samples except for sample 4. This result supports a previous finding that following retinal injury elevated vitreous cavity HGF/SF levels are associated with increases in c-Met expression and activation. Our results now suggest that the increase in c-Met receptor expression is a consequence of a decline in miR-182 expression that is needed for HGF to enhance RPE cell proliferation and migration.

## Discussion

As there are at least 2,000 different miRNAs regulating the expression of a large number of coding genes in humans, it is a technical challenge to identify the miRNA cohort in a specific tissue regulating a response contributing to the pathogenesis of a specific pathological condition. Thus in this study, we first target and focus on miR-182 in PVR development based on our previous study [22]. We found in PVR specimens that miR-182 expression is dramatically downregulated relative to its level in normal RPE cells obtained from individuals who had no evidence of PVR. As this effect in RPE cells was associated with increases in their proliferation and migration, miR-182 downregulation could precede PVR development. Such an association is tenable because these increases were lessened by upregulating miR-182 expression in RPE cells.

In PVR, RPE cells constitute a large proportion of the different cell types within the fibrocellular membranes in the vitreous cavity [4, 6]. Accordingly, they were used to delineate the role of miR-182 expression level changes in mediating molecular events leading to phenotypic changes similar to those associated with PVR. There have been only a few studies describing the roles of miRNAs in their posttranscriptional inhibition of gene expression in RPE cells. Specifically, these studies described: a) miR-204/211 involvement in maintaining the RPE phenotype [23]; b) miR-34a maintaining RPE cells quiescent and stationary [18]; c) differentially expressed miRNAs in TGFβ-induced EMT in RPE cells [24], and miR-124 involvement in this process [25]; e) the role of miR-23a [26] and miR-184 [27] in age-related macular degeneration. However, to the best of our knowledge we are not aware of any reports describing any involvement of a change in miRNA expression in clinical PVR tissue samples.

During the development of PVR, blood borne cytokines gain access to RPE cells due to retinal detachment breaching the BRB. They can induce through activation of their cognate receptors increases in RPE cell proliferation and migration during dedifferentiation [7, 28]. HGF/SF is one of the numerous cytokines in the vitreous chamber from PVR patients whose levels increase and contribute to the rises in RPE cell proliferation and migration [15, 16]. TGFβ2 levels within the retina and the vitreous also increase inducing RPE cells to dedifferentiate into mesenchymal type cells (EMT) which embed themselves within the forming fibrocellular membranes [29–32].

HGF/SF interacts with its cognate receptor, c-Met, which undergoes upregulation as it is phosphorylated by its intrinsic tyrosine kinase activity [33]. C-Met activation triggers through stimulation of downstream signaling pathways involving ERK1/2 and PI3K marked increases in cell migration whereas the increases in cell proliferation are less pronounced. The contribution by HGF/SF to eliciting these responses was confirmed by showing that knockdown of c-Met siRNA in RPE cells diminished these rises. Our result is also supported by an independent study that inhibitors targeting on PI3K/AKT signaling pathway can effectively suppress the proliferation of RPE cells [34]. The contribution by HGF/SF to increasing RPE migration in patient samples is indicated by our finding that c-Met expression was markedly elevated in PVR tissue samples relative to that in patients whose retinal detachment was not complicated by PVR.

Despite these findings, there are still two limitaions of this study: a. the small size of PVR samples, to some extent, impedes generalization of our findings; b. it is more likely that multiple miRNAs, rather than a single one are involved in PVR process. A global analysis of miRNAs with a large PVR sample size would facilitate addressing the above issues in subsequent studies.

Taken together, we demonstrate that miR-182 expression is downregulated in PVR, which leads to its target gene c-Met undergoing upregulation. This rise contributes to an increase in RPE cell proliferative and migratory activity through augmentation of the PI3K/Akt signalling pathway. This insight suggests that developing novel agents which can selectively upregulate miR-182 expression, or targeted delivery of miR-182 mimic to RPE, may have therapeutic value in improving the management of complications caused by PVR.

## Materials and Methods

### RPE cell isolation and culture

Human RPE layers were isolated from four adult donor eyes and cultured as previously described [35]. In brief, the anterior segment, vitreous, and retina of four donor eyes were excised first. Then a layer was immersed in a trypsin-EDTA solution at 37°C for one hour. The dispersed tissue was isolated and collected under direct observation using a dissecting microscope. Isolated RPE cells from three donors were directly used for RNA extraction. At the same time, the isolated RPE cells from the other remaining donor were centrifuged, resuspended, and seeded to a culture dish. Primary RPE cells were cultured in F12 medium (F12; Invitrogen, Carlsbad, CA) supplemented with 10% fetal bovine serum (FBS; Hyclone, Logan, UT) and 2 mM glutamine and incubated at 37°C in a humidified incubator containing 5% CO_2_. HEK-293 cells were purchased from ATCC (Manassas, VA).

### Ethics statement

PVR specimens were collected from patients treated at the First Affiliated Hospital, Zhejiang University (Hangzhou, China). Sample collection was approved by the Zhejiang University Ethics Committee on research involving human subjects, and written informed consent was obtained from each case. All experiments were performed in compliance with the Helsinki Declaration and national laws.

### Quantitative RT-PCR

Total RNA (10 ng) extracted from cells with TRIzol^®^ reagent (Invitrogen) was used for cDNA synthesis by the Taqman^®^ MicroRNA Reverse Transcription Kit (Applied Biosystems, Foster City, CA), and miR-182 expression level was quantified with the Taqman MicroRNA Assay (Applied Biosystems), according to manufacturer’s instructions. Real-time RT-PCR was performed using the Applied Biosystems 7500 Fast Real-Time PCR System (Applied Biosystems). The expression of miR-182 was normalized to the expression of U6 small nuclear RNA, and relative expression levels were calculated as previously reported [36].

### Cell proliferation assays

RPE cells, plated at 3 x 10^3^ cells per well in 96-well plates (Costar, High Wycombe, UK) were transfected with 50 nM of miR-182 precursor molecule (Ambion, Austin, TX) using Lipofectamine 2000 (Invitrogen). After 24-h culture, cell proliferation was assessed using the CellTiter 96 AQueous assay kit (Promega, Madison, WI) according to the manufacturer’s instructions. C-Met specific siRNA (Ambion) and its negative control siRNA (Ambion) were used for the transfection, which was followed 3 days later by the MTS assay.

### Flow cytometry analysis

RPE cells were first transfected with 50 nM miR-182. After 48 h, the cells were collected, washed with PBS, and stained with propidium iodide using the Cycle Test Plus DNA Reagent Kit (Becton Dickinson, San Jose, CA). The stained cells (1 x 10^5^) were then analyzed for DNA content with a flow cytometer (FACScaliber, Becton Dickinson).

### Transwell migration assays

RPE cells were grown to ~60% confluence and transfected with 50 nM of each miRNA. After 24 h, the cells were harvested by trypsinization and subjected to cell migration and transference studies. To measure cell migration and transference, 8-μm pore size-culture inserts (Transwell; Costar) were placed into 24-well culture plates. In both experiments, 400 μL of F12 containing recombinant human HGF (20 ng/mL; R&D Systems, Minneapolis, MN) were added to the lower chamber and 1 x 10^5^ cells were then added to the upper chamber. After 24 h of incubation, the number of cells that had migrated through the pores was quantified by counting ten independent visual fields under the microscope (Zeiss, Oberkochen, Germany) using a 20X objective.

### Luciferase reporter assays

The 3' UTR of human c-Met was amplified from human genomic DNA and individually cloned into the pMIR-REPORT vector (Ambion) by directional cloning. Seed regions were mutated to remove all complementarity to nucleotides 1–7 of miR-182 by using the QuickchangeXL Mutagenesis Kit (Stratagene, La Jolla, CA). HEK-293 cells were co-transfected with 0.4 μg of firefly luciferase reporter vector and 0.02μg of the control vector containing *Renilla* luciferase, pRL-SV40 (Promega), using Lipofectamine 2000 (Invitrogen) in 24-well plates (Costar). Luciferase gene reporter assays were performed 24 h after transfection using the Dual Luciferase Reporter Assay System (Promega).

### Western blot analysis

RPE cells (1 x 10^5^), grown in F12 with 10% FBS for 24 h, were transfected with miR-182 or negative control. Western blot analyses were performed as previously reported [22]. Antibodies for total ERK1/2, phosphorylated-ERK1/2, total Akt, phosphorylated-Akt, phosphorylated-Rb, c-Met and phosphorylated-Met were from Cell Signaling Technology (Beverly, MA).

### Statistical analysis

All data are shown as the mean ± SEM. Differences were analyzed using the Student’s t-test. Statistical significance was accepted at *P* < 0.05.
